# Invasive ductolobular carcinoma of the breast: spectrum of mammographic, ultrasound and magnetic resonance imaging findings correlated with proportion of the lobular component

**DOI:** 10.1186/2193-1801-2-621

**Published:** 2013-11-20

**Authors:** Gisela LG Menezes, Maurice AAJ van den Bosch, Emily L Postma, Mary–Ann El Sharouni, Helena M Verkooijen, Paul J van Diest, Ruud M Pijnappel

**Affiliations:** Department of Imaging, University Medical Centre Utrecht, E01.132, P.O. Box 85500, 3508, GA Utrecht, The Netherlands; Department of Surgery, University Medical Centre Utrecht, PO Box 85500, 3508, GA Utrecht, Netherlands; Department of Pathology, University Medical Centre Utrecht, PO Box 85500, 3508, GA Utrecht, Netherlands

**Keywords:** Breast carcinoma, Lobular, Ductal, Mammography, Ultrasonography, Magnetic resonance imaging

## Abstract

**Purpose:**

The aim of this study was to describe the imaging features of patients with invasive ductolobular carcinoma of the breast in comparison with the proportion of the lobular component.

**Materials and methods:**

We retrospectively reviewed mammographic, sonographic and MRI records of 113 patients with proven ductolobular carcinoma diagnosed between January 2008 and October 2012 according to the BI-RADS ® lexicon, and correlated these to the proportion of the lobular component.

**Results:**

At mammography the most common finding (62.9%) for invasive ductolobular carcinoma was an irregular, spiculated and isodense mass. On ultrasound an irregular and hypoechoic mass, with spiculated margins and posterior acoustic shadowing was observed in 46.8% of cases. Isolated mass and mass associated with non-mass like enhancement (NMLE) were the most common findings by MRI (89.4%). Washout pattern in delayed phase was seen in 61.2% and plateau curve was more frequently observed in patients with larger lobular component. Additional malignant findings (multifocality, multicentricity and contralateral disease) did not correlate significantly with the proportion of the lobular component.

**Conclusion:**

Invasive ductolobular carcinoma mainly presents as an irregular, spiculated mass, isodense on mammography and hypoechoic with posterior acoustic shadowing. On MRI it is usually seen as an isolated mass or as a dominant mass surrounded by smaller masses or NMLE. Washout is the most ordinary kinetic pattern of these tumors. In general, the imaging characteristics did not vary significantly with the proportion of the lobular component.

## Introduction

Breast cancer is a heterogeneous group of tumors with multivariate morphology, growth pattern, molecular profiles and response to treatment. The majority of invasive breast cancers (72 – 80%) are categorized as invasive ductal carcinoma (IDC). The prevalence of the second most common type of breast cancer, invasive lobular carcinoma (ILC), accounts for 5 to 15% (Biglia et al. 2007; Li et al. 2003; Verkooijen et al. 2003).

There is an extensive literature on clinical and imaging characteristics of both IDC and ILC (Acs et al. 2001; Arps et al. 2013; Brem et al. 2009; Kim et al. 2011; Korhonen et al. 2004; Lopez & Bassett 2009; Sastre-Garau et al. 1996; Winston et al. 2000; Yang et al. 2007). Multiple differences in demographic and tumor features between these two histological types have been reported. Patients with ILC are generally older at the time of the diagnosis, (Sastre-Garau et al. 1996; Moran et al. 2009) ILC is usually larger in diameter, (Arpino et al. 2004; Biglia et al. 2013) is more frequently hormone receptor positive, (Arps et al. 2013; Arpino et al. 2004) has lower grade than IDC, (Arps et al. 2013; Arpino et al. 2004; Biglia et al. 2013) is more frequently multifocal, multicentric and bilateral, and the organ distribution of metastatic disease tends to spread to pelvic organs, gastrointestinal tract and also to distinct sites such as retroperitoneum, meninges, ovary and serosa.

ILC has the histological characteristic to spread in rows of single cell layers around normal ducts like a “spider web”, infiltrating the preexisting stroma without inducing a strong desmoplastic response (Michael et al. 2008; Qureshi et al. 2006). This growth pattern causes minimum disruption of the normal anatomical structures than IDC, turning the radiological and clinical diagnostic of this tumor into a real challenge (Yeatman et al. 1995). This insidiously invasive nature makes the full extent of these tumors difficult to diagnose in screening. Mammogram may only reveal subtle changes or can even be completely normal. Mammographic sensitivity for detection of ILC varies between 57-92% (Butler et al. 1999; Hilleren et al. 1991a; Le et al. 1992) and ILC has higher false negative rates than IDC (19 vs. 10%) (Framarino Dei et al. 1995), making ILC more difficult to diagnose, especially in early stage. Ultrasound (US) is slightly more sensitive than mammography (sensitivity between 68-95%) and has shown to be more accurate in determine the (pathologic) size of the lesion, and also in identifying multifocality and multicentricity (Butler et al. 1999; Berg et al. 2004; Chapellier et al. 2000; Paramagul et al. 1995; Selinko et al. 2004). Magnetic resonance imaging (MRI) has become mainstream for diagnosis and work-up of breast cancer patients and many studies have demonstrated this imaging modality to have sensitivity above 90% (Butler et al. 1999; Berg et al. 2004; Chapellier et al. 2000; Boetes et al. 1995; Boetes et al. 1997; Harms et al. 1993; Mumtaz et al. 1997; Orel et al. 1994; Orel & Schnall 2001; Peters et al. 2008; Qayyum et al. 2002; Rodenko et al. 1996). MRI plays a fundamental role in providing additional information not obtained by conventional digital mammography and ultrasound, being of great importance in recognition of ipsilateral and contralateral lesions (Rodenko et al. 1996; Mann et al. 2008). The MONET trial demonstrated that breast MRI was associated with an increased re-excision rate and is not advised to be used routinely for preoperative work-up of patients with non-palpable breast cancer (Peters et al. 2011). However, various authors proposed preoperative breast MRI to have significant impact in treatment of patients with ILC (Kim et al. 2011; Lopez & Bassett 2009; Michael et al. 2008; Qayyum et al. 2002; Mann et al. 2008; Boetes et al. 2004; Schelfout 2004). MRI has a superior accuracy (Berg et al. 2004; Boetes et al. 2004) in defining the extent of ILC and is, therefore, essential for a correct surgical planning and further treatment of these patients (Boetes et al. 1995; Orel et al. 1994; Rodenko et al. 1996; Mann et al. 2008; Peters et al. 2011; Lesser et al. 1982).

Invasive ductolobular carcinoma, also called invasive ductal carcinoma with lobular features (IDC-L), is intermediate in the histological spectrum from ILC to IDC, but the clinical and radiological presentation and behavior of this histological type have not been widely studied. It is therefore not well known if imaging features, clinicopathologic behavior, and outcome of these tumors are more comparable to IDC or to ILC.

The aim of this study was to therefore describe the spectrum of mammographic, sonographic and MRI features according to the BI-RADS® lexicon in patients with histologically proven invasive ductolobular carcinoma of the breast and to evaluate the relationship between the proportion of the lobular component and the imaging characteristics of these breast tumors.

## Methods

### Patients

Patients diagnosed with invasive breast carcinoma containing lobular features at the UMC Utrecht (The Netherlands) between January 2008 and October 2012 were considered. Only patients who underwent pre-operative MRI, mammography and US were included in this study.

### Imaging acquisition

For the mammograms, the standard craniocaudal view and mediolateral oblique were obtained using the Hologic Lorad Selenia full field digital mammography system. Additional views or spot compression were obtained when necessary. The US images were acquired using a Philips HD-11 XE digital imaging system (5–12 MHz linear probe).

The MRI scans were acquired with the patient in the prone position on 3 Tesla clinical MRI scanners (Achieva, Phillips Healthcare, Best, The Netherlands) equipped with dedicated phased-array bilateral breast coils (SENSE-Breast7TX and SENSE-Breast-4 MRI devices).

MRI imaging was performed according to our standard staging breast imaging protocol, which included a transverse high-resolution T1-weighted isotropic volume examination (THRIVE) [TE/TR 1.87/4.9 ms; flip angle 10°; FOV 360 × 360 × 180 mm^3^, acquired voxel size 0.65 × 0.65 × 2.0 mm^3^, reconstructed voxel size 0.64 × 0.64 × 1.00 mm^3^) and a transverse SPAIR T2-weighted series (TE/TR 100/5508 ms; inversion delay SPAIR 305 ms; flip angle 90°; FOV 360 × 360 × 180 mm^3^, acquired voxel size 1.00 · 1.46 · 2.0 mm^3^, reconstructed voxel size 0.64 · 0.64 · 2.00 mm^3^). The dynamic series consisted of contrast-enhanced fat-suppressed T1-weighted gradient echo images (TE/TR 1.24/3.3 ms; flip angle 10°; FOV 360 · 360 × 180 mm^3^, acquired voxel size 1.00 · 1.00 · 2.00 mm^3^, reconstructed voxel size 0.94 · 0.94 · 1.00 mm^3^; dynamic scan duration 68 seconds). Images were acquired before and at 0, 69, 138, 206, 274 and 342 seconds after the administration of 0.1 mmol/kg Gadolinium-DTPA (Magnevist, Schering, Germany). The acquisition time of this scan package was approximately 25 minutes.

### Image interpretation

Mammograms and US images were retrieved from the local PACS system and analyzed at a Sectra Workstation IDS7 (Sectra Imtec AB, Sweden). MRI examinations were processed by CADstream (Confirma, Inc., Kirkland, WA, USA). The images were interpreted by two dedicated breast radiologists. In addition, all images were reviewed and interpreted by a third radiologist, who was blinded to the proportion of the lobular component of each patient. In case of discordance with the original reports, a consensus was reached with a fourth dedicated breast radiologist with more than 20 years of experience in breast imaging.

All images were interpreted according to the guidelines of the BI-RADS® lexicon (D’Orsi & Dea 2003). Lesions were essentially divided into mass and non-mass-like lesions in order to perform morphological analysis. Based on the BI-RADS® lexicon, (D’Orsi & Dea 2003) lesions which had a mass as the main characteristic [isolated mass or dominant mass surrounded by smaller masses or foci of non-mass like enhancement (NMLE)] were defined as a mass-like lesion. Architectural distortion and NMLE (focal area, linear, ductal, segmental, regional, multiple regions, diffuse enhancement, and multiple enhancing foci) were defined as descriptors of non-mass-like lesion. Time intensity curves were classified according to their pattern of initial rise (slow, medium, rapid) and according to the delayed phase (persistent, plateau, washout). Finally, each lesion was scored according to the BI-RADS® lexicon; (D’Orsi & Dea 2003) 0 – Finding for which additional evaluation is needed, 1 – No abnormal enhancement, no lesion found, 2 – Benign finding, 3 – Probably benign finding, (short interval follow-up), 4 – Suspicious abnormality, 5 – Highly suggestive of malignancy, 6 – Known cancer biopsy-proven malignancy diagnosis on the imaged finding prior to definitive therapy.

Tumor extent and additional disease were defined as follows:

- Multifocality: an additional malignant lesion in the same quadrant, separated from the index tumor by benign tissue.
- Multicentricity: an additional malignant lesion in a different quadrant than the index cancer.
- Contralateral disease: an additional malignant lesion found in contralateral breast.
- Multiplicity: two or more of these features: multifocality, multicentricity and contralateral disease.

Additional findings were considered true positives when histopathological analysis of either preoperative work-up or surgical specimen has shown malignancy [invasive carcinoma or ductal carcinoma in situ (DCIS)].

### Histological analysis

All slices of ductolobular carcinoma (n = 113) were reviewed by a dedicated breast radiologist to quantify the lobular component, defined as a proportion of the invasive cancer.

### Statistical analysis

Data were analysed using SPSS 17.0 software (SPSS, Inc., Chicago, IL, USA). Chi – square tests were used to compare proportions of the lobular component to the imaging characteristics and to compare the proportions of lobular component to the additional findings in our sample. For statistical purpose, proportion of the lobular component was grouped into three different categories: ≤ 20%, 21 – 94%, and ≥ 95%. Results were considered significant at *p < 0.05.*

## Results

Between January 2008 and October 2012, 505 patients were diagnosed with breast cancer and invasive ductal carcinoma with lobular features was reported in 30% (155/505) of the patients. Of these 155, 41 patients were excluded due to technical problems in performing MRI, obesity, claustrophobia, impossibility of obtaining mammography or ultrasound before MRI and personal reasons. The remaining 113 patients who underwent mammography, ultrasound and MRI were selected for this study. The age at diagnosis of the 113 patients ranged from 34 to 87 years with a mean of 57.4 years.

There were 41 patients with a proportion of ≤ 20% of lobular component, 36 patients with a proportion of 21 to 94%, and 36 patients with ≥ 95% of lobular component.

### Mammographic findings

Mammographic findings are presented in Table 1. A mass was the most common mammographic finding, observed in 54.8% of cases. In 46.0% of cases, we found an isolated mass and, in 8.8%, a mass was found associated with microcalcifications. Isolated microcalcifications were seen in 11.5% of the patients. Architectural distortion was found in 10.6% of cases and asymmetries (associated or not with microcalcifications) were noticed in 8.1% of cases. Normal findings were observed in 15.0% of the patients.

**Table 1:** **Mammographic findings of invasive ductolobular carcinomas of the breast**

Findings	n = 113
Benign/Normal	15.0% (17/113)
Mass	46.0% (52/113)
Mass with calcification	8.8% (10/113)
Calcification only	11.5% (13/113)
Architectural distortion	10.6% (12/113)
Focal asymmetry or asymmetry	7.1% (8/113)
Asymmetry and calcification	1.0% (1/113)
**Mass shape**	**n = 62**
Round/Oval	8.1% (5/62)
Lobular	3.2% (2/62)
Irregular	88.7% (55/62)
**Mass Margin**	**n = 62**
Circunscribed	4.8% (3/62)
Not Circunscribed	24.2% (15/62)
Spiculated	71.0% (44/62)
**Mass Density**	**n = 62**
Isodense	88.7% (55/62)
Hyperdense	11.3% (7/62)
**Associated Findings**	**n = 113**
Nipple retraction	3.5% (4/113)
Skin Thickening	10.6% (12/113)
Enlarged axillary lymph nodes	3.5% (4/113)

Prevalence of normal findings was higher (25.0%) in patients with ≥ 95% of lobular component vs. 12.2% in the ≤ 20% lobular component group (Table 2). However, this difference had no statistical significance *(p = 0.115).*

**Table 2:** **Imaging findings of invasive ductolobular carcinomas of the breast according to proportion of the lobular component**

	Proportion lobular component		
**Findings**	≤ 20%	21 – 94%	≥ 95%
	(n = 41)	(n = 36)	(n = 36)
Normal Mammographic Findings	12.2% (5)	8.3% (3)	25.0% (9)
Multifocality	21.9% (9)	27.7% (10)	33.3% (12)
Multicentricity	9.7% (4)	19.4% (7)	25.0% (9)
Contralateral Disease	9.7% (4)	11.1% (4)	16.6% (6)
Multiplicity	7.3% (3)	13.8% (5)	30.5% (11)

**Figure 1:**
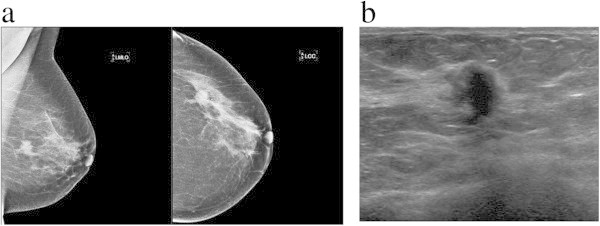
**48 year old woman, with positive family history for breast cancer, presented with a palpable lump on the left breast, finally diagnosed as ductolobular carcinoma with a **≤** 20% proportion of the lobular component. a)** Mediolateral oblique and craniocaudal mammograms show an irregular and spiculated mass. A comet-tail like projection arises from the anterior and posterior margins and a discrete retraction around the lesion can be seen. **b)** Ultrasound of the left breast demonstrates an irregular, hypoechoic and vertically oriented mass, with echogenic halo at 3 o’clock position.

Considering only mass lesions (n = 62), a total of 62.9% were simultaneously irregular, spiculated and isodense to the fibroglandular parenchyma (Figure 1a). Isodense mass was more frequently associated with smaller lobular component (*p = 0.016*). Circumscribed masses were seen in 2.6% (3/113) of the patients.

The prevalence of other mammographic lesions (microcalcifications, architectural distortion, asymmetries), mass shape and mass margins did not vary significantly according to the proportion of the lobular component.

**Figure 2:**
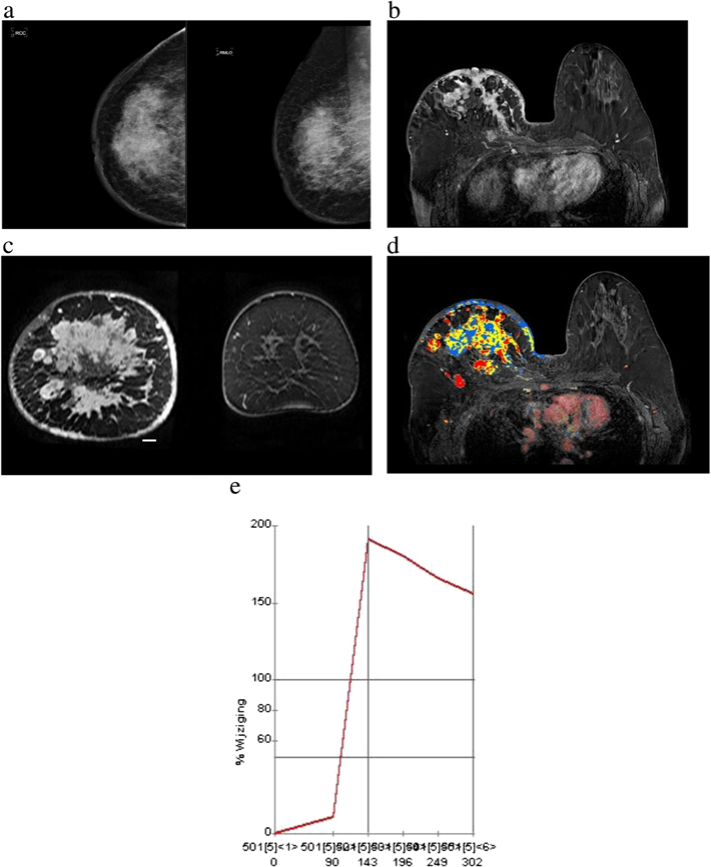
**59 year-old patient with mastitis carcinomatosa (inflammatory carcinoma), finally diagnosed as ductolobular carcinoma with a **≥** 95% proportion of the lobular component. a)** Craniocaudal and mediolateral mammograms show extensive skin and trabecular thickening/coarsening of the right breast and diffusely increased breast density, associated with multiple calcifications in the upper outer breast. **b)** MRI shows multiple confluent masses, with irregular margins and heterogeneous internal enhancement pattern. Skin thickening and invasion of the nipple and the pectoral muscle are also observed. These are the typical features of inflammatory carcinoma. Multicentricity (multifocal and multicentric disease simultaneously) can also be observed in coronal projection **(c)**. **d** and **e)** Post-processed color parametric map image demonstrates multiple areas of malignant enhancement in axial projection. Kinetic curve demonstrates typical malignant pattern (rapid initial rise and washout).

**Figure 3:**
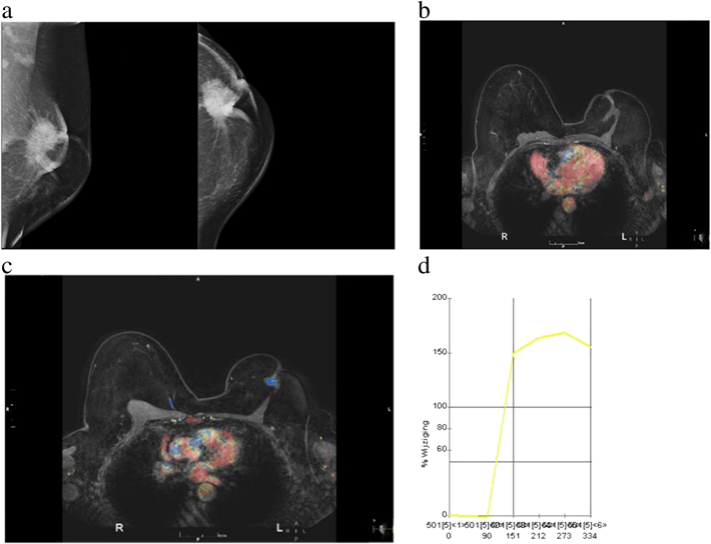
**52 year-old woman with nipple retraction and metastasis to the right eye, finally diagnosed as ductolobular carcinoma with a 21 – 94% proportion of the lobular component. a)** Left mediolateral oblique and craniocaudal mammograms show a star shaped mass in center of the breast, associated with calcifications, skin thickening and nipple retraction. **b)** Axial contrast material-enhanced with fat suppression show a spiculated mass. There is nipple retraction, skin thickening and pectoral muscle invasion. **c** and **d)** Post-processed color parametric map and kinetic curve predominantly demonstrate persistent enhancement and a small central area of plateau.

Additional findings (nipple retraction and skin thickening) were seen in 14.1% (16/113) of the patients (Figures 2a and 3a).

### Ultrasound findings

**Figure 4:**
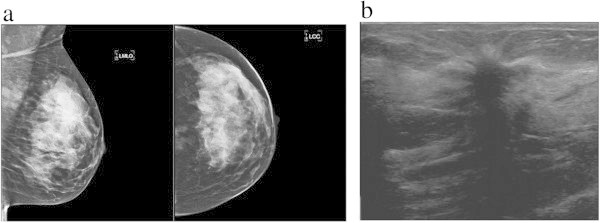
**A 43 year old woman presenting with focal thickening in the left breast, finally diagnosed as ductolobular carcinoma with a **≤ **20% proportion of the lobular component. a)** Mediolateral and craniocaudal mammograms of the left breast show a spiculated mass in the upper outer quadrant. **b)** Ultrasound shows a hypoechoic and spiculated mass, with echogenic halo at and posterior acoustic shadowing (upper outer quadrant).

Ultrasound findings are summarized in Table 3. Of the 113 cancers, 109 (96.5%) lesions were masses localized in the breast, 3 (2.6%) patients had normal exams and 1 (0.9%) patient had a parasternal mass. These masses (n = 109) found in our study were mainly irregular (92.7%), spiculated (60.6%), hypoechoic (91.8%), with posterior acoustic shadowing (64.2%). These four characteristics were observed simultaneously in 46.8% of the patients (Figures 1b and 4b). Microlobulated margin was seen in 19.3% of the patients and absence of posterior acoustic features was found in 30.3% of cases.

**Table 3:** **Ultrasonographic findings of invasive ductolobular carcinomas of the breast**

Findings	n = 113
Benign	2.6% (3/113)
Mass	96.5% (109/113)
Other findings (parasternal mass)	0.9% (1/113)
**Mass Shape**	**n = 109**
Round/Oval	7.3% (8/109)
Irregular	92.7% (101/109)
**Mass Margin**	**n = 109**
Circumscribed	1.8% (2/109)
Indistinct	10.0% (11/109)
Angular	8.3% (9/109)
Microlobulated	19.3% (21/109)
Spiculated	60.6% (66/109)
**Mass Echogenicity**	**n = 109**
Complex echoic	6.4% (7/109)
Hypoechoic	91.8% (100/109)
Isoechoic	1.8% (2/109)
**Mass Posterior acoustic feature**	**n = 109**
No feature	30.3% (33/109)
Enhancement	5.5% (6/109)
Shadowing	64.2% (70/109)
**Associated Findings**	**n = 113**
Enlarged axillary lymph nodes	8.8% (10/113)

Taking into account the lobular component, angular margins were more prevalent in patients with a bigger lobular component, with 11.1% in the ≥ 95% group and 4.8% in the ≤ 20% group. However, these results had no statistical significance. Findings concerning to mass shape, echogenicity and posterior acoustic features had similar prevalence in all groups.

### MRI findings

Of all tumors (n = 113), 89.4% (101) were classified as “mass-like” lesion (see Table 4). From these 101, 56.4% were isolated masses, 37.6% were a dominant mass associated with NMLE features and 6.0% were dominant masses surrounded by smaller masses. The most common findings for mass lesions were low signal on T1 in 99.0% of cases and moderate signal in 43.6% of cases on T2-weighted images. Concerning mass shape, the masses found were predominately irregular (86.1%). Regarding the margins of these masses, spiculated or irregular margins were found in 91.1%. Heterogeneous enhancement pattern was seen in 59.4% of cases.

**Table 4:** **MRI findings of invasive ductolobular carcinomas of the breast according to the BI-RADS® (NMLE = non-mass like enhancement)**

Lesion Type	n = 113
Normal	1.8% (2/113)
Mass^a^	55.8% (63/113)
Mass + NMLE	33.6% (38/113)
NMLE	5.3% (6/113)
Architectural distortion	2.6% (3/113)
Architectural distortion + NMLE	0.9% (1/113)
**Mass T1**	**n = 101**
Low	99.0% (100/101)
Mod	0% (0/101)
High	0% (0/101)
High central low peripheric	1.0% (1/101)
**Mass T2**	**n = 101**
Low	7.9% (8/101)
Mod	43.6% (44/101)
High	37.6% (38/101)
Low central high peripheric	10.9% (11/101)
**Mass Shape**	**n = 101**
Round/Oval	6.0% (6/101)
Lobular	7.9% (8/101)
Irregular	86.1% (87/101)
**Mass Margin**	**n = 101**
Smooth	8.9% (9/101)
Irregular	36.6% (37/101)
Spiculated	54.5% (55/101)
**Mass Enhancement**	**n = 101**
Homogeneous	15.8% (16/101)
Heterogeneous	59.4% (60/101)
Rim enhancement	24.8% (25/101)
Dark internal septation	0% (0/101)
Enhancing internal septation	0% (0/101)
Central enhancement	0% (0/101)
**NMLE Distribution**	**n = 45**
Focal area	71.1% (32/45)
Linear	11.1% (5/45)
Ductal	4.5% (2/45)
Segmental	2.2% (1/45)
Regional	2.2% (1/45)
Multiple Regions	6.7% (3/45)
Diffuse	2.2% (1/45)
**NMLE Internal Enhancement**	**n = 45**
Homogeneous	69.0% (31/45)
Heterogeneous	22.2% (10/45)
Stippled, punctate	0% (0/45)
Clumped	4.4% (2/45)
Reticular, dendritic	0% (0/45)
Rim enhancement	4.4% (2/45)
**NMLE Symmetry**	**n = 45**
Symmetric	0% (0/45)
Asymmetric	100.0% (45/45)
**Kinetic Pattern Initial Rise**	**n = 113**
Not available	7.1% (8/113)
Slow^b^	0% (0/103)
Medium^b^	13.6% (14/103)
Rapid^b^	86.4% (89/103)
**Kinetic Pattern Delayed Phase**	**n = 113**
Not available	7.1% (8/113)
Persistent^b^	6.8% (7/103)
Plateau^b^	32.0% (33/103)
Washout^b^	61.2% (63/103)

^a^ Isolated mass or dominant mass surrounded by smaller masses.

^b^ Excluding non-available and normal exams (n = 103).

39.8% of patients presented with NMLE features and they could be found isolated or associated with other lesions (adjacent to a dominant mass or to an architectural distortion). Considering all cases with NMLE features found in our sample (45 in total), 71.1% presented as focal areas. The internal enhancement was homogeneous in 69.0% and all cases were asymmetric.

Lesions with isolated NLME aspect were seen in 5.3% (6/113) of our patients. Architectural distortion and architectural distortion associated with NMLE were seen in 2.6% and 0.9% of the patients, respectively. Normal exams were found in 2 patients (1.8%).

**Figure 5:**
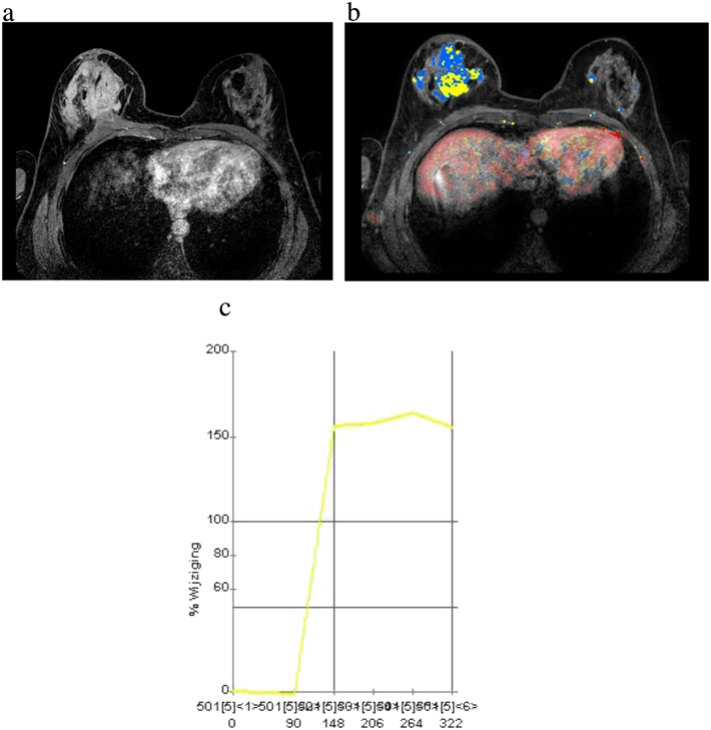
**49 year-old patient with palpable thickening in the right breast, finally diagnosed as ductolobular carcinoma with a** ≤ **20% proportion of the lobular component. a)** Multiple irregular, confluent and heterogeneous masses in the right breast associated with skin thickening and pectoral muscle invasion. **b** and **c)** Contralateral disease is better seen in color parametric map (axial projection). Observe the enhancement pattern (rapid initial rise and plateau) commonly observed in invasive lobular carcinomas.

Considering all lesions with available kinetic data (n = 103), rapid initial rise was seen in 86.4% of cases and in 61.2% of cases had washout pattern in delayed phase. In 7.1% of cases, kinetic data was not available and 6.8% of the patients showed benign pattern (persistent curve) in delayed phase. In kinetic delayed phase, “plateau” curve was more frequently observed in cases with bigger lobular component (36% in both the 21 – 94% and ≥ 95% groups vs. 17% in the ≤ 20% group) (Figures 3c, d and 5c). Washout was more prevalent in tumors with a smaller lobular component (63.4% in the ≤ 20% group vs. 50.0% in the ≥ 95% group) (Figure 2e) . Despite these differences, there was no statistical relevant difference among those groups (*p ≥ 0.05*). Mass shape, mass margins, patterns of mass enhancement and NMLE characteristics did not show statistical significant variations according to the proportion of the lobular component.

We found 46 associated findings in 36 patients (31.8%), such as nipple retraction, skin thickening (focal or diffuse), edema, hematoma/blood and pre-contrast high ductal signal. Invasion of the pectoral muscle, confirmed by histopathological analysis, was found in 5 (4.4%) patients, nipple invasion in 3 (2.6%) patients and skin invasion in 2 (1.8%) cases. Chest wall invasion was seen in 1 (0.9%) patient, associated to pectoral muscle ingrowth and, in 2 (1.8%) cases, nipple, skin and pectoral muscle ingrowth were found together (Figures 2b, c, d, 3b and 5a).

### Lymphadenopathy

Lymph node metastasis was histologically reported in 57 patients. From these, 38.5% were seen by imaging. In 5 cases, lymphadenopathy was seen both in MRI and US and in 3 cases it was seen in MRI, US and mammography. Prevalence of lymphadenopathy did not show significant differences according to proportion of the lobular component.

### Additional findings

Multifocality and Multicentricity were found in 31 (27.4%) and 20 (17.7%) patients respectively. Multiplicity was found in 19 cases (16.8%) and contralateral disease was seen in 14 (12.3%) patients (Figure 2c and 4b). Taking into account the proportion of lobular component, contralateral disease, multifocality and multicentricity had higher prevalence rates in patients with bigger lobular component (Table 2), but there was no statistical significance. Nevertheless, multiplicity was more likely to be found in patients with a bigger lobular component (*p = 0.021*).

### Size

At mammography and US, tumor size ranged from 4 to 6.0 cm (mean, 1.78 cm) and on MRI tumor size ranged from 2 to 9.4 cm (mean, 2.50 cm). The sizes found on pathology ranged from 2 to 12.0 cm (mean, 2.9 cm).

## Discussion

### Mammography

The characteristics of IDC and ILC have been extensively described in literature. The dispersed infiltrating growth pattern of ILC with very little desmoplastic reaction and, consequently, the development of palpable lesion or tumors detectable in imaging exams, is less frequent. Normal or benign findings are more common in ILC than in IDC (8%–16% vs. 1.1%) (Li et al. 2003; Kim et al. 2011; Selinko et al. 2004; Mann et al. 2008) and false negative rates for ILC in mammography range from 14 to 19% (Kim et al. 2011; Hilleren et al. 1991b; Krecke & Gisvold 1993).

In the current study, we observed 15% (17/113) of normal or benign mammograms, which might demonstrate a similar behavior to ILC. It was also interesting that patients with tumors with ≥ 95% of lobular component had a higher prevalence of normal findings in mammography than patients with ≤ 20% of lobular component (25 vs. 12%, respectively), but these differences were not statistically significant.

ILC is usually seen as a mass (44%–65% of cases), (Lopez & Bassett 2009; Helvie et al. 1993) having predominantly irregular and spiculated margins (63-71%) (Helvie et al. 1993; Evans et al. 2002) and is usually isodense when compared to the fibroglandular tissue (Kim et al. 2011; Lopez & Bassett 2009; Sickles 1991). These numbers were similar to our present findings. Mass was found in 54.8% of our sample and 62.9% of these masses were simultaneously irregular, spiculated and isodense.

ILC spreading diffusely through the breast stroma leads to lower tendency to form round and circumscribed masses, only seen 1%–3% of cases of ILC (Le et al. 1992). The lobular component of ductolobular tumors might lead to a similar behavior and, in our study, circumscribed masses were indeed only found in 2.6% of our cases.

Architectural distortion was seen in 10.6% of cases and asymmetries were found in 7.9% of cases. Literature findings refers 10%–16% of ILC cases manifesting as architectural distortion (Hilleren et al. 1991b; Helvie et al. 1993) and 4%–13% of cases expressing as asymmetries (Hilleren et al. 1991b; Helvie et al. 1993). Our findings are comparable to these ILC mammographic lesions. However, architectural distortion is the second most common finding in ILC (Hilleren et al. 1991b;Helvie et al. 1993) and architectural distortion was the third most common radiological abnormality in our study.

It is well known that microcalcifications are much less common when comparing ILC and other breast carcinomas (4-24% vs. 41%) (Le et al. 1992). The prevalence of microcalcifications in our study was similar to the referred prevalence of these findings in ILC (11.5%).

### Ultrasonography

US is considered more sensitive than mammography in detecting ILC. Literature reports sensitivities ranging from 68 to 98% (Paramagul et al. 1995; Selinko et al. 2004) and this imaging modality is also more accurate in identifying multifocality, multicentricity and size of the lesion (Selinko et al. 2004). According to Kim and Butler, mass has been described as being the most common lesion found in US in cases of ILC (60.5 – 100%) and both authors agreed that an irregular, hypoechoic mass, with spiculated margins and posterior acoustic shadowing is the most ordinary pattern seen in US images of ILC (Kim et al. 2011; Butler et al. 1999). Our results are consistent with these numbers. However, Kim et al. described US features of ILC and IDC as being very similar, except for posterior acoustic features, which has been described as being more related to ILC (Kim et al. 2011).

### MR imaging

MRI has proven to have a high overall sensitivity (approximately 95%) (Mann et al. 2008; Kneeshaw 2003) and, in adjunct to mammography and US, has an essential importance in diagnostic and staging of ILC. MRI has a moderate specificity (67.4%) (Bluemke et al. 2004) and the routinely clinical use of this imaging modality might lead to unnecessary procedures (Peters et al. 2011). However, in ILC cases, MRI is superior to other imaging modalities in estimating tumor size, detecting multifocality, multicentricity, contralateral disease (Boetes et al. 1995; Orel et al. 1994; Rodenko et al. 1996; Mann et al. 2008; Peters et al. 2011; Lesser et al. 1982). and also affecting surgical management in 28% of cases (Mann et al. 2008; Weinstein 2001). Mass is considered the most common manifestation of ILC at MRI and the incidence varies substantially (45%–95%) (Kim et al. 2011; Rodenko et al. 1996; Schelfout 2004; Weinstein 2001; Yeh et al. 2003). Most of these studies have not described ILC findings in MRI strictly according to the BI-RADS® lexicon. However, Hye Na Jung and Kim found 92% and 88.8% of ILC cases presented as mass - like lesion according to the BI-RADS® (Kim et al. 2011; Jung et al. 2013). These findings are consistent with our research (89.4% of cases presenting as mass - like tumors).

In a literature review, Mann et all described 85.5% (65/76) of ILC tumors presenting as an irregular or spiculated mass (Mann et al. 2008). Our study had similar results and 91% (92/101) of the lesions were described as irregular or spiculated masses.

T1 and T2 features of lobular tumors are not frequently mentioned in literature. Levrini reported 95.2% (20/21) of cases of ILC tumors being hypo- and hyperintense lesions on T2 weighted TSE images (Levrini et al. 2008). Unfortunately all of our patients underwent diagnostic procedures within 10 days before MRI. The hemorrhage, edema and necrosis that result from these procedures may have changed T1 and/or T2 signal, which makes an accurate analysis more difficult.

Not many studies refer to the kinetic behavior of ILC. The infiltrative growth pattern of these tumors seems not to require extensive neovascularization and the lack of endothelial growth factor found in lobular tumors turns the new vessels to grow more slowly and having better maturation, resulting in less permeable capillaries (Lee et al. 1998). Some studies found ILC having delayed maximum enhancement and wash out pattern was not observed in the majority of tumors (Trecate et al. 2001; Sittek et al. 1998). These features might be due the histological behavior of ILC. Indeed, the prevalence of “plateau” curve in our study was higher in groups of patients with higher lobular component, and washout was more prevalent in groups of patients with lower lobular component. However, these differences were not statistically significant.

More recent studies refer 70.3% to 95.2% of ILC lesions having washout pattern (Kim et al. 2011; Levrini et al. 2008; Mann et al. 2011). Considering all available kinetic curves and not taking into account the proportion of the lobular component, washout was the most common pattern in our analysis (61.2%), but still lower than the numbers referred from these authors. However, Mann at al. showed that when CAD-application was used to evaluate the kinetic curve of lesions of ILC and IDC, washout pattern has a very similar prevalence in both tumors, which is not the case for visual assessment. In this latter case, IDC has a much higher prevalence of washout than ILC (Mann et al. 2011). The use of CADstream software to obtain the kinetic curves in our study might be the explanation for the higher washout pattern prevalence.

### Lymphadenopathy

Arps et al. described IDC-L as having a higher frequency of nodal metastasis when compared to IDC and ILC (51 vs. 34 and 45%, respectively) (Arps et al. 2013). A similar result was seen in our study and lymph node metastasis was found in 50.4% of our sample (57/113), even though there was no statistical significance between prevalence of lymphadenopathy and proportions of lobular component.

### Additional findings

Since comprehensive studies about mixed tumors are missing in literature, it is difficult to put our present results into perspective. Arps et al. compared clinicopathologic features and outcomes of 183 cases of IDC-L with lobular features with 1499 patients with IDC and 375 patients with ILC. The authors concluded that the clinicopathologic features and outcomes of IDC-L and ILC are very similar, irrespective of the proportion of the lobular component (Arps et al. 2013).

In our study, not only imaging characteristics did not vary significantly according to the lobular component, but also multifocality, multicentricity, contralateral disease and the proportions of lobular component did not show a statistically significant correlation.

However, the significant association between two or more of these additional findings (multiplicity) and bigger lobular component (*p = 0.021*) is in line with the higher rates of additional disease foci in patients with ILC (Boetes et al. 1995; Orel et al. 1994; Rodenko et al. 1996; Mann et al. 2008; Peters et al. 2011; Lesser et al. 1982).

## Conclusion

To our knowledge, this is the first study to exclusively describe radiological features of invasive ductolobular carcinoma. They typically present as an irregular, spiculated and isodense mass at mammography, as a hypoechoic, irregular and spiculated mass with posterior acoustic shadowing on US, and as an isolated mass or as a dominant mass surrounded by smaller masses or NMLE on MRI. Washout is the most ordinary kinetic pattern of these tumors. Except for isodensity and multiplicity, the imaging characteristics did not vary significantly according to proportion of the lobular component. The imaging features and the high incidence of additional malignant imaging findings of invasive ductolobular carcinoma are therefore more similar to ILC than to IDC.

### Ethical standards

The study complies with current Dutch legislation.
